# What are the Critical Elements of Satisfaction and Experience in Labor and Childbirth—A Cross-Sectional Study

**DOI:** 10.3390/ijerph17249295

**Published:** 2020-12-12

**Authors:** Barbara Baranowska, Anna Kajdy, Paulina Pawlicka, Ernest Pokropek, Michał Rabijewski, Dorota Sys, Artur Pokropek

**Affiliations:** 1 Department of Midwifery, Centre of Postgraduate Medical Education, 01-813 Warsaw, Poland; barbara.baranowska@cmkp.edu.pl; 2 Department of Reproductive Health, Centre of Postgraduate Medical Education, 01-813 Warsaw, Poland; mirab@cmkp.edu.pl (M.R.); dsys@cmkp.edu.pl (D.S.); 3 Department of Cross-Cultural and Gender Psychology, Institute of Psychology, University of Gdansk, 80-309 Gdansk, Poland; paulina.pawlicka@ug.edu.pl; 4 Institute of Telecommunications, Warsaw University of Technology, 00-661 Warsaw, Poland; er.pokropek@gmail.com; 5 Educational Research Institute (IBE), 01-180 Warsaw, Poland; artur.pokropek@gmail.com

**Keywords:** labor, experience, perinatal care, cross-sectional studies, Poland

## Abstract

The labor experience and satisfaction with childbirth are affected by the care provided (external factors) and individual variables (internal factors). In this paper, we present a descriptive analysis that aims to indicate the strongest correlates of birth experience among a wide range of indicators. The study is a prospective, cross-sectional, self-report survey. It includes the experiences of women giving birth in public and private hospitals in Poland. The two main variables were birth experience and satisfaction with care. The analysis consists of three parts: data pre-processing and initial analysis, explorative investigation, and regression analysis. Among the 15 variables with the highest predictive value regarding birth experience were being informed by the medical personnel, communication, and birth environment. The most significant variables among 15 variables, with the highest predictive value regarding care, were those concerning support, information, and respectful care. The strongest predictor for both, birth experience and satisfaction with care, is the sense of information, with logit coefficients of 0.745 and 1.143, respectively, for birth experience and satisfaction (0.367 and 0.346 for standardized OLS coefficient). The findings demonstrate that by using explanatory variables, one can predict a woman’s description of her satisfaction with perinatal care received in the hospital. On the other hand, they do not have such a significant and robust influence on the birth experience examined by the variables. For both the birth experience and satisfaction with care, the sense of being informed is the highest predictor.

## 1. Introduction

Women’s birth experience is an essential aspect of the evaluation of perinatal care [1]. As such, labor constitutes an intricate and intimate process that can be satisfying and fulfill a woman’s expectations, but at times might be harmful and even traumatic. A positive experience is a birth that meets or exceeds one’s own beliefs and socio-cultural expectations. A healthy baby is born, and a woman receives both reliable emotional support and professional care delivered by competent clinical personnel. Additionally, a woman’s positive experience includes a need for natural labor, providing a sense of personal achievement and control through decision-making, even when medical interventions are needed [2]. There are many tools available to evaluate and define the childbirth experience [3]. A concept analysis carried out by Larkin et al. described it “as an individual life event, incorporating interrelated subjective psychological and physiological processes, influenced by social, environmental, organizational and policy contexts” [4].

The labor experience and satisfaction with childbirth are affected by the care provided (external factors) and individual variables of the woman in labor (internal factors) [5,6,7,8,9]. The quality of the care provided includes conditions of the facility, support, and communication with medical personnel. Individual variables are the woman’s traits, such as coping with childbirth, preparedness, and relationship with the baby [10].

The medical literature contains the concepts of positive and negative, and even traumatic birth experience [11,12,13]. In qualitative research conducted by Karlström et al. [11], a characteristic of a positive birthing experience is described as being related to self-empowerment (the confidence of being able to bring life into the world) as well as the company and support of people assisting during labor [11].

A systematic overview of women’s needs that specified essential aspects of childbirth demonstrated that most healthy women of reproductive age desire a positive experience and safety and the inclusion of labor’s psychological context [14]. The latest WHO guidelines, Intrapartum care for a positive childbirth experience, stress the need to include the expectations of women, since this, in addition to science-based medical care, contributes to creating a positive childbirth experience [2].

Medical personnel has limited control over the aspects of childbirth-related to individual traits, the course of labor, or the health of the baby. It can, however, improve the quality of care, thus shaping the childbirth experience. The improvement of women’s satisfaction in the perinatal period constitutes a challenge for care systems for the woman and child. This study of women’s experiences responds to current research objectives “Action for research to improve the quality of care for every woman, every child” [15]. Prior research points out that birth experience is related to combinations of many factors, and there were no attempts to comprehensively study their correlations.

This study aims to determine and analyze the most influential and potent ones, which could later serve the hospital staff as leading indicators for possible improvement of the birth experience and guide future research addressing it.

## 2. Materials and Methods

### 2.1. Design and Setting

The study is a prospective, cross-sectional, self-report survey. It includes the experiences of women giving birth in the years 2017–2018, in public and private hospitals in Poland. The study was conducted in February–March 2018 using an online self-administered survey questionnaire. The survey was designed to minimalize the fatigue of the respondents. The average time of response was 20 min. The design of the survey allows to fill the questionnaire in multiple attempts. Information about the survey appeared on social media as well as in the press and on the radio. Interested women were asked to provide their email address, consent to data processing, and receive our newsletter. Activation links to the survey page were sent to the email addresses submitted.

Eight thousand three hundred seventy-eight surveys qualified for analysis. In the study of results, data were weighted according to information from the General Statistical Bureau. We resorted to edge weighting according to the region (voivodship) and residence of the mother (city vs. countryside).

The questionnaire contained 122, mostly closed-ended questions, and five questions about socio-demographic variables (Supplementary Materials). The survey also asked about experiences in the admissions office during childbirth, and maternity ward stay.

The two main variables were birth experience and satisfaction with care. Respondents qualified the birth experience in five ways: as an ecstatic experience; source of great pleasure, and empowerment; as a positive experience; as something one must simply get through; as a negative experience, like a traumatic experience, the most horrible event imaginable. This was followed by a question ranking satisfaction with care from 1 to 5, one being poor, and five very good.

Other variables included in the study concerned preparation for childbirth (participation in childbirth classes, having a birth plan), childbirth process (the type of labor, methods of relieving pain, interventions applied), course of postpartum care (help with feeding and caring for the child), and patient-personnel communication (means of communication, conveying information and obtaining permission for procedures).

### 2.2. Statistical Analyses

The analysis consists of three parts: data pre-processing and initial analysis, explorative investigation, and regression analysis. For the first part, we preselected 140 variables; the selection was based on general theoretical assumptions. Preselected variables concern demographical information, antenatal factors (e.g., participation in childbirth classes, having a birth plan), childbirth process (e.g., type of childbirth, methods of relieving labor pain), child’s condition, staff behavior during childbirth with particular emphasis on communication.

Using such a large number of variables as 140 in the classical multivariate analysis is not recommended due to collinearity, estimation, and interpretation problems. Therefore, in the second part, we performed an exploratory analysis to choose the most relevant variables for each childbirth experience, employing the random forest technique [16] for this task. This machine-learning algorithm was designed to perform accurate predictions considering the direct effects of variables and interactions between variables and nonlinear relationships. It has proven to be extremely useful with performance similar to neural networks or support vector machines [17,18,19]. What is unique in this technique is that variable importance, the measure of impact on each variable’s prediction, could be easily computed and used for variable selection [20,21]. The algorithm has proven to be more effective than classical methods, such as backward or forward stepwise regression, providing a higher ability to distinguish relevant from irrelevant variables and best perform final models [22] and therefore chosen for this research.

Our application random forest models consisted of 100 decision tree predictors, of which each output is considered for the final prediction. Scikit-learn’s default hyperparameters were chosen to create predictive models. Using the discrepancy of the classifications, optimal split conditions were found, measured by the Gini index [23]. The final prediction was based on the “most popular” one, i.e., the most occurring in the decision trees.

For the random forest analysis, we decided to merge two categories: ecstatic (8.34%) with positive (29.03%) experience, as well as negative (7.42%) and traumatic (9.72%) for the personal experience, which results in a total of three categories. We also collapse response category insufficient (1.92%) and poor (5.92%), which finally yields four categories in a question referring to satisfaction with care. This was done only in the explorative analysis using random forest which allows us to boost the performance for this approach.

In the final step, based on the 15 most relevant variables for each experience, we formulated the ordinal regression model [24] accompanied by classical ordinal last squares regression (OLS). The models were used to investigate the complex relationship between explanatory variables and outcome variables in more depth. While the ordinal regression model provides a better fit to the model by respecting the categorical nature of outcome variables and therefore provides the more valid significance, testing the coefficients is challenging to interpret. They are not directly comparable across models and samples (see [25] for an overview). To facilitate the interpretation and comparability of models, we are also presenting standardized coefficients from OLS regression that could be easily used as comparable measures of effect sizes. The regression analysis was performed using Stata software, while random forest using Stata 14.2 (StataCorp, College Station, TX, USA), as well as Python’s machine learning library sci-kit-learn.

### 2.3. Ethical Approval

The study received the approval of the bioethical committee of the Warsaw Medical University AKBE/232/2017.

## 3. Results

The largest group of respondents (44%) was in the 20–26 age bracket, and 97% had a high school or university degree. Most respondents (98.3%) were cared for by a gynecologist during their pregnancies; 65.6% had their first babies; 90% had a person accompanying the childbirth, while 67% had participated in childbirth classes. Natural childbirth accounted for 61.6% and cesarean section for 35.8% of births Table 1.

The childbirth experience was most commonly described as “hard to describe as positive or negative, or as a positive experience.” (Table 2).

Most respondents evaluated their stay in the maternity ward well or very well. (Table 2).

### 3.1. Most Predictive Variables

The Random forest procedure resulted in an accuracy of around 51.27% for the birth experience and about 67.64% for satisfaction. The predictors had a strong tendency to pick the most occurring classes (mostly middle); however, this complexity of the data techniques, such as oversampling or synthetic data generation, has not shown any notable improvement in metrics. The importance of metrics is presented in the figures below. We have chosen the 15 variables with the highest predictive value and importance score higher than 1.5%.

**Figure 1:**
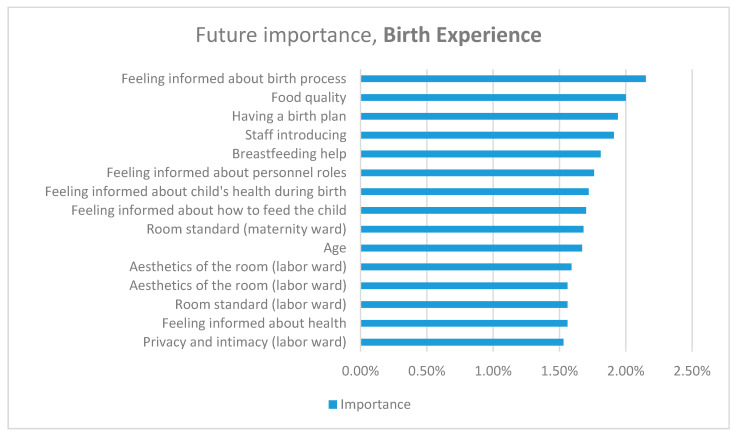
Most predictive variables for birth experience.

The 15 variables with the highest predictive value regarding birth experience were being informed by the medical personnel, communication, and birth environment. The guarantee of intimacy and privacy in the labor room; equipment, the standard, and aesthetic quality of the labor room; and information given to the woman on health matters were rated significantly at the level of 1.53–1.56%. (Figure 1) Considered important at the level of 1.59–1.76% were variables that describe the equipment, standard, and aesthetic quality of the maternity ward, and the mother’s age. Importance at the level of 1.70–1.81% referred to feeling informed about the baby’s health, feeding the baby, and breastfeeding. The staff introduces themselves, the quality of food, and having a birth plan rated at 1.91–2.0%. The most prominent variable (2.15%) describes the need to be informed about the childbirth process (Figure 1).

**Figure 2:**
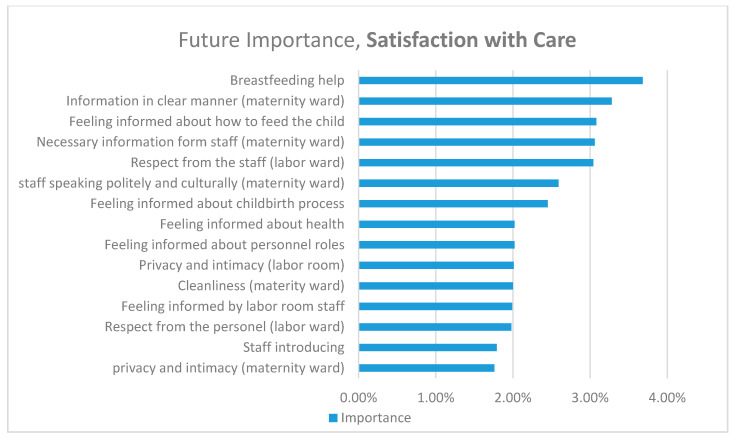
Most predictive variables for satisfaction with care.

The most significant variables among 15 variables, with the highest predictive value regarding care, were those concerning support, information, and respectful care. Variables concerning privacy and intimacy during the hospital stay; respect shown to the woman in the labor ward; equipment, aesthetic quality, and standard of the labor room; staff introductions and presentation of clear roles for personnel; and information concerning health and cleanliness of the maternity ward were considered significant at 1.76–2.02% (Figure 2). Information about the childbirth process, polite communication by the personnel, and respect shown to the woman reached a 2.45–3.04% level. Clear communication of necessary information by medical personnel, such as feeding the child, reached a level of importance of 3.06–3.28%. Evaluation of support in the area of lactation had the highest predictive value of 3.68%. (Figure 2).

### 3.2. Variable Selections and Construction of the Composite Indices

We recognized that the set of variables describing a general sense of information was picked by random forest analysis based on the explorative analysis. We have constructed a scale consisting of variables from the seven questions: “In the hospital, did you feel sufficiently informed about: the childbirth process, your health, health of the child during childbirth, newborn’s health, how to feed your child, procedures performed on the child, who was your lead doctor/midwife?” The scale proved to have high reliability of 0.89 (as measured by Cronbach’s alpha), showing that items could be aggregated in a reliable composite measure. The scale is simply a standardized sum of scores from item respondents, where 0 indicates insufficient information received from staff and four full information.

The summarized scale was also prepared for the maternity ward’s standard of rooms (Cronbach’s alpha 0.86). It was based on five questions regarding the evaluation of conditions after birth in the following categories: “cleanliness, the standard of equipment (e.g., cabinets, beds), room standard (e.g., bathroom, size), aesthetics of the department (e.g., decor, interior colors), and food quality”. A general assessment of obstetric/midwifery staff from the maternity ward (Cronbach’s alpha 0.89) was based on six questions: “Did the maternity ward staff show you respect, care for your privacy and intimacy, communicate with you in a cultured and well-mannered way, gave you all the needed information, gave you the information comprehensibly, knock before entering the room?” The explanatory analysis convinced us to also construct a general index for standards in the labor room (Cronbach’s alpha 0.8807). It was based on four questions concerning assessment of the labor room conditions in the following terms: “cleanliness, the standard of equipment (e.g., cabinets, beds), room standard (e.g., bathroom, size), and aesthetics of the ward (e.g., decor, interior colors)”. Furthermore, we constructed the general index for assessment of labor room staff (Cronbach’s alpha 0.93), based on four questions: “Did the labor room staff show you respect, care for your privacy and intimacy, communicate with you in a cultured and well-mannered way?” The last two indices could be used only for women with the labor room and its staff.

Apart from the series of variables that indicate the quality of information, staff, and material standard, four additional variables were suggested by explanatory analysis. Information about breastfeeding (How do you assess breastfeeding support?), whether the staff were introducing themselves, and whether women had a birth plan and their women. The last three were coded as binary variables, while the others were standardized and treated as continuous.

### 3.3. Regression Analysis

We started examining the results by analyzing R2, which denotes the percent of variance explained for each model for each outcome variable (Table 3). It is immediately apparent that used variables explain satisfaction with care much better than birth experience. Pseudo R2 from the ordered model for satisfaction with care was 0.060, while birth experience yielded 0.377; that is more than six times higher and more than four times higher according to the standardized coefficient from the OLS model.

The strongest predictor for both dependent variables is the sense of information, with logit coefficients of 0.745 and 1.143, respectively, for birth experience and satisfaction (0.367 and 0.346 for standardized OLS coefficient). The variable describing the obstetric/midwifery staff has only a slightly smaller effect for satisfaction with a logit coefficient of 0.989 and a standardized OLS coefficient of 0.326. In comparison, this variable’s impact is much weaker for birth experience (logit coefficient of 0.098 and standardized OLS coefficient of 0.05). The same pattern could be found for the variable responsible for the standard of rooms (maternity ward) with ordered logic coefficients 0.093 and 0.590, respectively, for birth experience and satisfaction (0.046 and 0.168 for standardized OLS coefficient model).

Breastfeeding has a different relation to the birth experience and satisfaction with care: −0.111 for overall birth experience and 0.484 in ordered logit models (−0.056 and 0.484 for OLS models with standardized coefficients). The negative coefficient for information on breastfeeding is unexpected. The bivariate correlation between breastfeeding and birth experience information is positive and is equal to 0.20 (the corresponding correlation for satisfaction with care is 0.57). Negative effects occur when we are controlling for other variables that are also correlated with information on breastfeeding (e.g., sense of information Cor = 0.59; obstetric/midwifery staff Cor. = 0.55), resulting in a situation where having all other factors fixed, an additional piece of information yields a prediction of a slightly worse birth experience.

A birth plan did not show a robust direct effect in the regression analysis (non-significant results at *p* < 0.01 for all models). However, as this variable was selected by random forest procedure, its effect might not be direct, or the variable could serve as a moderator to other variables, which needs some further analysis.

Interestingly, if the staff was introducing themselves, a single variable reporting had a powerful impact on satisfaction according to ordered logit, with a statistically significant coefficient of 0.509 (not confirmed, however, by regression). No significant relation was found for the overall experience. The relation for age is not strictly linear. The lowest birth experience was observed in women aged 26–35 (negative and statistically significant coefficients at *p* < 0.05) while younger and older women show higher grading of the experience. As for the satisfaction with care, women at age 31–35 are, on average, the most satisfied, mothers younger than 26-yers old the least, while mothers of age 26–30 and more aged than 36 are between the groups mentioned above.

Table 4 demonstrates results for a group of women, excluding women not in the labor room due to the cesarean procedure. For this group, we could add two additional variables: Standards in the labor room and labor room staff to confront it with room standards in the maternity ward and obstetric/midwifery staff. In the previous table, many women (1640, which is almost 20% of the sample) did not visit labor rooms and could not express their opinion about it.

## 4. Discussion

This paper presents a descriptive analysis of the strongest correlates of birth experience among a wide range of indicators, including demographics, antenatal factors (e.g., participation in childbirth classes, having a birth plan), mode of childbirth (e.g., childbirth process, type of childbirth, methods of relieving labor pain), neonatal outcome, or staff behavior during childbirth (with particular emphasis on communication).

Satisfaction with care was described by over 50% as high or very high. These results are similar to other studies on Polish women in reproductive age. Stadnicka et al.’s studies show that 50.7% of women questioned were satisfied with midwifery care during their time in the labor room [26]. Similarly to studies of Kraśnianin et al., 8 percent of respondents judged their perinatal care to be poor or very poor [27]. This stands in contrast to results obtained in Sweden, where only 2.6 percent of respondents evaluated their care during birth as poor or very poor.

This is an exciting finding showing how important the labor room staff is for birth experience. Additionally, our study results highlight the need to be informed and included in decision-making expressed through a desire to receive information. These results align with WHO recommendations, where being informed and having staff support are essential variables for a positive birth experience [2].

In our study, receiving information was one of the most important predictors of both positive experience and a high level of satisfaction. The importance of being informed in the context of a positive birth experience has also been shown in studies by Mukamurigo et al. [28]. Additionally, researchers point to ineffective communication and lack of information as an obstacle to having a positive childbirth experience [29]. Perhaps, studies should be designed to research risk factors of ineffective communication both for the patient and medical personnel.

Our results show that elements of medical services qualifying as respectful care have predictive value. Women list respect and privacy as essential aspects of childbirth experience and satisfaction with perinatal care. Earlier studies of perinatal care in Poland show that respectful care is lacking. Consequently, many women may find it challenging to achieve a positive birth experience due to a lack of respectful care. The need to educate medical personnel in respectful care becomes even more critical given our research [30]. Education of medical personnel should be audited prospectively to allow better understanding of factors affecting respectful care.

Having a birth plan could act as a mediating factor in the birth experience. However, our study did not show a strong correlation between having a birth plan and a positive birth experience. Studies show that the fulfillment of expectations marked out in the plan, not the possession of the plan itself, had a considerable impact [31,32].

Breastfeeding has a different relation to the birth experience and satisfaction with care. The correlation between information on breastfeeding and birth experience is positive. Negative effects occur when we are controlling for other variables that are also correlated with information on breastfeeding. In this situation, having all other factors fixed, an additional piece of information on breastfeeding for patients results in a prediction of a slightly worse birth experience. This might be interpreted casually, bearing in mind that the highest level of birth experience is defined as “Childbirth was an ecstatic experience, a source of pleasure and empowerment.” Having all other factors fixed, an additional surplus piece of breastfeeding information might cause rather unexpected consequences. More likely, however, some not stringboard patterns of interaction among investigated attributes exist, and the role of information is not straightforward, being possibly mediated by the effects of other variables (see [33,34]). Whether support in breastfeeding can modify the birth experience is a valuable research question.

In our study, the type of birth (vaginal vs. cesarean section) did not show high predictive value for satisfaction with care or birth experience. This is contrary to Swedish studies that have demonstrated the negative effect of cesarean section [35].

Our results show that the maternity ward and labor room environment strongly impact women’s satisfaction and experiences. The environment was defined as the aesthetics, comfort, and cleanliness of the rooms. This is according to the results of international studies that have highlighted environmental factors as very significant predictors of maternal satisfaction [36]. Hammond and Foureur’s research considers the environment as an essential element of shaping birth culture, impacting its quality and the woman’s experience. The birth environment has been previously described as a unique domain included in the model of interconnectivity [15,37]. According to this model, the space in which the birth occurs forms women’s experience and behavior. In Swedish studies, the birth environment constituted a critical aspect of influencing satisfaction with care [35].

The quality of the labor room was important for the birth experience. Additionally, both obstetric/midwifery staff and labor room staff contributed equally to the satisfaction with care. The standards of rooms in the maternity ward have a more substantial effect on satisfaction with care. This component of satisfaction is believed to have a massive impact on the effectiveness of the staff’s care and attitudes [38].

An interesting finding was made when comparing women with a labor ward experience versus women with a cesarean section without the labor ward experience. In general, all previous relations observed for all women remained the same. It became clear that the labor room staff’s quality was much more critical for the birth experience than obstetric/midwifery staff. On the other hand, both obstetric/midwifery staff and labor room staff contributed equally to care satisfaction. When controlling for both obstetric/midwifery staff and labor room staff, the rooms’ standards lose the direct impact on the general birth experience, becoming insignificant to both maternity ward and labor room. Standards of rooms in the maternity ward have a more substantial effect on assessing satisfaction with care, respectively.

The lowest birth experience was observed in women aged 26–35, while younger and older women show higher grading of the experience. As for the satisfaction with care, women at age 31–35 are the most satisfied, and mothers younger than 26 years old are the least. The majority of reports did not find any correlation between the woman’s age and the satisfaction with care during and after childbirth [26,36,39,40]. Mothers in the age group 25–29 and 30–34 years were more likely to declare high satisfaction with care than those aged 15–19 years [41]. This correlation, unprecedented in previous studies, between specific age groups, birth experience, and satisfaction requires further research to determine the reasons for these differences.

Based on the variables related to perinatal care described by women, we selected the factors that best predict birth experience and satisfaction of care. The experience and satisfaction studied by us differed in terms of correlates. It is immediately apparent that used variables explain satisfaction with care much better than birth experience. This makes much sense as most of the analysis variables describe factors that should be connected with the quality of service, thus theoretically should be closely related to satisfaction. However, the overall birth experience is probably something more individual and driven by additional sets of women’s predispositions not measured in this study. The satisfaction with care could be directly linked to the factors describing the quality of service, while the overall experience is probably a more complex phenomenon.

The direction of future research should incorporate qualitative studies of the birth experience, taking into account women with different expectations and needs (e.g., including millennials or women on the autism spectrum) to investigate this phenomenon in more detail. Since information is so important for patients that it is necessary to look at various of communication in relation to women’s various needs in childbirth and the puerperium.

The study’s limitation is having obtained data only from questionnaires, from women who volunteered for the study. Moreover, the time that had passed since giving birth (it could have been a maximum of 1 year and three months after giving birth) could have influenced women’s memories. Our research is a correlation analysis. A controlled experiment should be performed. Our results can be used to design such an investigation, pointing to the most interesting potential determinants.

One of the limitations of the study is the fact that it was based on a detailed self-administered online questionnaire. The main disadvantage of self-administration online surveys is that they might select more digital literacy respondents introducing selection bias. However, in the second decade of 21st century, in developed countries, we expect this effect to be minor. The other potential problem of such studies is the self-administration nature of the questionnaire, where respondents could not ask for question of clarification or could not rely on help when some technical problems occurred. On the other hand, self-administered questionnaires are less susceptible to information bias (for example, social desirability bias) and interviewer effects [42] and hence might be better for evaluation purposes. Finally, our questionnaire involved many detailed questions with significant collection burden on respondents, which could result in careless responding or dropout. We tried to minimize these problems by designing the questionnaire to last approximately 30 min, which locates it as medium length online questionnaire [43] and ensures that the response rate will not be a major issue [44]. To shorten the survey, we utilized an advantage of electronic and web-based questionnaires that allow for automatic controlling of participant responses and administrating only questions relevant to previous answers. Overall, we believe that the adapted by us design shows a reasonable trade-off between cost-effectiveness and potential problems resulting in acceptable measurement of phenomena under investigation.

One of the most important nuances regarding machine learning models is their interpretability. The concept of feature importance has been utilized to see which features have the highest influence of the prediction, which may help us to understand how the model makes decisions. Although this approach has gained huge attention in recent years, it is not without limitations that we have to consider. Strobl et al. illustrate that if the dataset contains features of vastly different types, magnitudes, or categories, the feature importance analysis might yield biased and sometimes even misleading results [45]. Furthermore, reliable analysis of the random forest requires fine tuning and at least satisfying predictive power of the model, and even in this case, when the number of features is too high (compared to the size of the dataset), their importance might not be informative enough, given their small magnitudes. In such case, those parameters would require further statistical investigation, as it was performed in this study.

The impact of breastfeeding support on the birth experience is not clear and requires further analysis. Our method does not allow for an estimate on whether breastfeeding (a predictive aspect for birth experience) was perceived by women as support in the labor room, during stage 3 of birth or general support in lactation throughout the hospital stay.

The variables used in the study correlate more clearly with satisfaction than with experience. Therefore, to learn about the key elements influencing the birth experience, it is necessary to conduct additional studies considering other variables.

## 5. Conclusions

The findings demonstrate that by using explanatory variables, one can predict a woman’s description of her satisfaction with perinatal care received in the hospital. Additional research on the birth experience is needed, considering other variables, such as a woman’s personality traits, attitudes towards childbirth, and obstetric history. Both for the birth experience and satisfaction with care, the sense of being informed is the highest predictor. Our results strongly suggest that providing information about breastfeeding can significantly increase satisfaction with care. Although necessary for both experience and satisfaction, the evaluation of the obstetric/midwifery staff has a higher impact on satisfaction with care.

## Figures and Tables

**Table 1:** The sociodemographic description (*n* = 8378).

Age	M = 29 Me = 30	
**Place of Residence**	***n***	**%**
Village	3418	41
City < 50 000inhabitants	1109	13
City 50,000–100,000 inhabitants	805	10
City 100,000–500,000 inhabitants	1313	16
City > 500,000 inhabitants	1733	21
**Education**	***n***	**%**
Primary	87	1
Secondary trade education	130	2
High school	1716	20
Higher education	6445	77
**Financial status**	***n***	**%**
Very low		0
Low		0
Average		23
Good		56
Very good		20

* M= average; Me = median.

**Table 2:** Distribution of answers corresponding to expressions responses qualifying the birth experience and ranking the satisfaction with care (*n* = 8378).

	The Content of the Answer	*n*	%
**Responses qualifying** **the birth experience**	Childbirth was a traumatic experience, the most horrible event I have ever experienced	814	9.72
	Childbirth was a negative experience	622	7.42
	Childbirth was something one must get through	3811	45.49
	Childbirth was a positive experience	2432	29.03
	Childbirth was an ecstatic experience, a source of pleasure and empowerment	699	8.34
**Responses ranking the satisfaction with care**	1-Insufficient	161	1.92
	2-Poor	496	5.92
	3-Average	1557	18.58
	4-Good	3135	37.42
	5-Very good	3029	36.15

**Table 3:** Regression analysis for all respondents (*n* = 8378).

	Birth Experience				Satisfaction Form Care			
	Ordered Logit		OLS		Ordered Logit		OLS	
	Coef.	*p* > |*t*|	Beta	*p* > |*t*|	Coef	*p* > |*t*|	Beta	*p* > |*t*|
Sense of information	0.745	0.000	0.367	0.000	1.143	0.000	0.346	0.000
Standard of rooms (maternity ward)	0.093	0.000	0.046	0.000	0.590	0.000	0.168	0.000
Obstetric/midwifery staff	0.098	0.001	0.050	0.001	0.989	0.000	0.326	0.000
Information on breastfeeding	−0.111	0.000	−0.056	0.000	0.484	0.000	0.126	0.000
Birth plan	0.094	0.025	0.017	0.093	0.049	0.306	−0.002	0.726
Introduction of staff	0.038	0.582	0.004	0.702	0.509	0.000	0.005	0.467
Age (compared to less than 26)								
26–30	−0.163	0.022	−0.028	0.097	0.158	0.045	0.024	0.030
31–35	−0.155	0.032	−0.022	0.193	0.354	0.000	0.051	0.000
36 and more	−0.079	0.392	−0.001	0.910	0.331	0.002	0.027	0.002
PseudoR2/R2/	0.060		0.155		0.377		0.637	
*n*	8275		8275		8275		8275	

* OLS = Ordinary least squares.

**Table 4:** Regression analysis excluding women that were not in the labor room (*n* = 6738).

	Birth Experience				Satisfaction from Care			
	Ordered Reg		OLS		Ordered Reg		OLS	
	Coef.	*p* > |*t*|	Beta	*p* > |*t*|	Coef.	*p* > |*t*|	Beta	*p* > |*t*|
Sense of information	0.480	0.000	0.233	0.000	0.799	0.000	0.224	0.000
Standard of rooms (maternity ward)	0.042	0.170	0.022	0.138	0.448	0.000	0.118	0.000
Obstetric/midwifery staff	−0.020	0.569	−0.007	0.683	0.830	0.000	0.252	0.000
Information on breastfeeding	−0.069	0.021	−0.036	0.012	0.554	0.000	0.143	0.000
Birth plan	0.097	0.034	0.017	0.120	−0.086	0.104	−0.007	0.306
Introduction of staff	0.018	0.809	0.002	0.852	0.463	0.000	0.004	0.586
Age (compared to less than 26)								
26–30	−0.154	0.044	−0.027	0.132	0.050	0.565	0.008	0.488
31–35	−0.149	0.057	−0.024	0.184	0.221	0.013	0.030	0.008
36 and more	−0.045	0.671	0.003	0.847	0.209	0.089	0.014	0.114
Standards in the labor room	0.0358	0.225	0.0131	0.352	0.229	0.00	0.069	0.000
Labor room staff	0.5366	0.000	0.2535	0.000	0.7539	0.000	0.243	0.000
R2/PseudoR2	0.0755		0.1934		0.3996		0.5644	
*n*	6738		6738		6738		6738	

## Supplementary Materials

The following are available online at https://www.mdpi.com/1660-4601/17/24/9295/s1.
