# Supplementary material for: What are the Critical Elements of Satisfaction and Experience in Labor and Childbirth—A Cross-Sectional Study
Source: Int J Environ Res Public Health. 2020 Dec 12;17(24):9295. doi: 10.3390/ijerph17249295 (PMC7764244; doi:10.3390/ijerph17249295)
Supplement: Supplementary file 1 [file ijerph-17-09295-s001.pdf]

PAGE 1 – HOSPITAL

Question
**(9) I have not delivered yet** (Special question)
How many times did you deliver? *<br>• Once<br>• Twice<br>• Three times<br>• Four or more<br>• I have not delivered yet
**(5) Year of birth of child/youngest child**
**(133) Place of childbirth /youngest child** (special question)

PAGE 2 – BEFORE LABOR

Question content
**(8) Have you attended birthing class or have met with a midwife for prenatal education?** *
● Yes, in the last pregnancy<br>● Yes, in a previous pregnancy<br>● No
**(10) Who has managed your last pregnancy?**
● Obstetrician that worked in the hospital where I delivered<br>● Obstetrician that did not work in the hospital where I delivered<br>● Midwife that worked in the hospital where I delivered<br>● Midwife that did not work in the hospital where I delivered
**(11) What were the main criteria for choosing the hospital where you delivered your last child (please name 2 most important criteria)** *
● I chose the hospital closest to home<br>● Good opinions about the obstetric department<br>● In the hospital there is a neonatal pathology unit (there was an option of getting help if needed)<br>● The doctor or midwife that managed my pregnancy works at that hospital<br>● I could hire a midwife with whom I could deliver<br>● The hospital reffered me to other women who delivered in this hospital<br>● I delivered my previous child/children in this hospital<br>● There are very good housing and sanitary conditions<br>● Availability of epidural anesthesia<br>● Other, which?<br>● I did not think about hodpital choices
(12) What sources of information did you use to learn about the hospital of your choice? (please give to main sources of infromaation)
● Opinions of other mothers<br>● Online sight Gdzierodzic.info (wheretodeliver.info)<br>● Other online sights/forums<br>● From social media (i.e. Facebook)<br>● From the doctor<br>● From the midwife<br>● Birthing class<br>● From family/friends<br>● Other, which?<br>● Hard to say

PAGE 3 – ADMISSION WARD

Question content

(89) Did you have a birthing plan? *

- Yes, I had a birthing plan before arrival at the hospital
- Yes, I received it and filled it in the hospital

- No, I did not have a birthing plan
- I do not remember
- Does not apply

(90) Did domeone discuss the birthing plan with you?

- Yes, midwife
- Yes, doctor
- No, no one discussed the birthing plan with me
- I do not remember

(91) Was the birthing plan respected?

- Yes, all of it was respected
- Yes, with justified exceptions
- Yes, but not all of it
- It was not respected
- Hard to say

(13) did someone at the hospital discuss with you your expectations regarding birth and care during delivery?

- Yes
- No
- I do not remember
- Does not apply

(14) in your opinion, before delivery did the emergency room staff: *

- Show you respect
- Care for your privacy and intimacy
- Communicate with you in a cultural and well-mannered way
- Gave you all the needed information
- Gave you the information so that it was understandable

- Definitely yes
- Rather yes
- Neither yes nor no
- Probably not
- Definitely not

(15) Was the gynecological examination in the emergency room a typical examination for you? *

- Yes
- No, it was extremely painful and unpleasant
- I did not have an internal examination at the emergency room
- I do not remember
- Not applicable

(16) Has something happened in the emergency room that you did not agree with or violated your sense of security? *

- Yes
- Do not
- I do not remember
- Not applicable

(92) What happened in the emergency room, which you did not agree with or have violated your sense of security?

(17) Did you have an enema in the hospital before delivery?*

- Yes
- No
- I do not remember
- Not applicable

(93) Was the enema done with your consent?

- Yes
- No
- I do not remember

(94) Did you have your crotch shaved in the hospital?
*

- Yes
- No
- I do not remember
- Not applicable

(95) Was the crotch shaved with your consent?

- Yes
- No
- I do not remember

(96) Was you delivery: *	
• Natural vaginal<br>• Operational vaginal (vaccum, forceps)<br>• Cesarean section	
	(149) Was the cesarean section:
	• Planned (decision about cesarean section was made during pregnancy or during hospitalization)<br>• Unplanned (decision about cesarean section was made during delivery)
	(19) Was your delivery very fast or sudden (you gave birth within about an hour after admission to the hospital)?
	• Yes<br>• No<br>• Hard to say<br>• Not applicable
	(20)<br>Was your delivery a high-risk birth? (health situation of a child or mother, e.g. high prematurity, gestational hypertension)?
	• Yes<br>• No<br>• I do not know<br>• Not applicable
	(21) Was you child born:
	• In good condition,<br>• did not require medical help<br>• Required medical help<br>• Dead

PAGE 4 – DELIVERY

Question content
**(22) Did you have an intravenous caniluation inserted (into the peripheral vein)? medicines**
• Yes, with my permission<br>• Yes, without asking for permission<br>• Yes, without asking for permission and despite my opposition<br>• Did not<br>• I do not remember
**(23) In what room did you give birth?**
• Single room<br>• Multi-person, but no other giving birth<br>• Multi-person, with other women<br>• Another situation, what?
**(24) Who accompanied you during delivery? (you can select more than one answer)**
• I gave birth alone<br>• Child's father<br>• Mother<br>• Sister<br>• friend<br>• doula<br>• Another person close
**(25) Were students of medicine or midwifery present at your delivery?**
• Yes, with my permission<br>• Yes, but I was not asked for permission<br>• Yes, despite my opposition<br>• No, I did not agree to their presence<br>• No they were not
**(26) Did you have an induction of labor? (puncture of amniotic sac, or vaginal gel used)**
• Yes<br>• No<br>• I do not know

(98) Were you asked for consent for induction of labor?

- Yes
- No
- I do not remember

(27) Did you have an oxytocin drip during delivery?

- Yes
- No
- I do not know

(99) Why were you given an oxytocin drip?

- To induce labor
- To speed up labor
- I do not know / I do not remember

(100) Were you asked for consent for this procedurę?

- Yes
- No
- I do not remember

(28) Have you been provided with information about the pain relief methods available in the hospital? (eg massage, ball, bath / shower, dolargan, epidural, gas)

- Yes, I have received enough information
- Yes, I received information, but in my opinion incomplete
- No, I have not received any information
- I do not remember

(137) In order to relieve the pain of delivery, have you used:

- Dolargan
- Epidural anesthesia (in the spine)
- Local anesthesia for stitching the crotch
- Inhalant gas
- TENS
- Walking, walking
- Massage
- Warm or cold compaction
- bath / shower
- Bag of sako, ball, ladders

- Yes

- I wanted to use, but it was not possible
- I did not need to
- I do not know
- Did not use

(30) How often do you feel you had an internal examination during delivery?

- Too often
- Just right
- Too rare
- It was not performed
- It's hard to say
- Not applicable

(31) Did anyone ask you for permission before doing an internal examination?

- Yes, every time
- Yes, but not every time
- I was not asked for permission at all
- I do not remember

(32) How often has the child's heart been monitored with the CTG device during labor?

- I had to lie all the time during the delivery without interruption
- Continuously, but I could move
- From time to time and then I had to lie down
- From time to time, but I could move
- I do not remember
- Not applicable

(33) Did you have epiziotomy during your delivery?

- Yes, with my permission
- Yes, without my consent
- Did not
- I do not remember
- Not applicable

(34) During the delivery, in the delivery room, could you:

- Walk / change position
- drink
- Eat

- Yes
- Did not

- I do not know

(35) During the delivery, in the delivery room, could you:

- Use the shower / bathtub
- Use helpful equipment such as a ball, sako bag
- Yes
- Did not
- I could not because there were medical contraindications
- I could not - in the room where I was there was no such possibility
- I do not know / I do not remember

(130) In the second stage of delivery (during the birth of the head) were you in position (if the position changed, please tick all the answers):

- Flat on the back
- In a semi-sitting position
- On the side
- Sitting
- Squatting
- On all fours
- Standing
- Another position, what?
- Not applicable

(37) Who was deciding about the positions in which you gave birth in the second stage of labor?

- I myself decided
- Midwife / doctor advised me a position that will be good for me and I decided on it
- The position was chosen by the midwife / doctor, I could not decide
- I do not remember
- Another situation, what?
- Not applicable

(131) During the active phase of the second stage of labor (please tick all the answers that match your situation):

- You were encouraged to follow your own need to push
- You were encouraged to breathe naturally during the active contractions
- You were ordered to bend your head to your chest
- You were told to bend your legs to the belly
- You have been asked to take in air, keep it for longer and push hard
- You have been ordered push like on a stool
- You were criticized for the way in which you push
- I was so anesthetized that I did not feel the contractions and was dependent on the instructions of the staff
- Another situation, what?
- I do not remember
- Not applicable

(39) Was your belly pressed on (kneaded) during delivery?
?

- Yes
- No
- I do not remember

(102) When was your belly pressed on?

- First stage of labor (during cervix dilatation)
- Second stage of labor (when pushing the baby out)
- After the baby was born
- I do not remember

(101) What was your belly pressed with?

- Hand
- Elbow
- With all the body, lying on me
- Something else, what?

(40) Did the naked child lie on your naked breasts / belly immediately after giving birth (skin-to-skin contact)?

- Yes
- No
- I do not remember

(103) How long did the "skin to skin" contact last?

- Very short - a few minutes
- Short (eg until examining and stitching the crotch, about 15-30 minutes)
- Average (over 30 minutes, but less than 2 hours)
- Long (2 hours or longer, until leaving the delivery room)
- I do not remember

	(104) Why the contact "skin to skin" was short?
	● The child had problems and had to be examined by a doctor / taken to a neonatal ward<br>● The child had to be weighed and measured<br>● The child had to be dressed<br>● Due to my bad mood, I was not able to have a child with me<br>● For another reason (what?)<br>● I do not know

(41) Where was the child's Apgar score assessed?

- Na moim brzuchu / piersiach
- In the neonatal examination stand
- W innym miejscu
- Nie wiem

(42) Were you given support during the first breastfeeding in the delivery room?

- Yes
- Yes, but I did not want to feed
- No, because I was separated from the child
- Was not
- I do not remember

(43) Did the delivery room Staff:

- Show you respect
- Care for your privacy and intimacy
- Communicate with you in a cultural and well-mannered way
- Gave you all the needed information
- Gave you the information so that it was understandable

- Definitely yes
- Rather yes
- Neither yes nor no
- Probably not
- Definitely not

(151) Please, assess the conditions in the delivery room in the following categories (1 is insufficient, 5 very good):

- cleanliness
- Standard of equipment (eg cabinets, beds)
- Room standard (e.g. bathroom, size)
- Aesthetics of the department (eg decor, interior colors)

PAGE 5 – CESAREAN SECTION

Question Content
**(44) Did you receive information about cesarean section surgery?**
• Yes, I have received enough information<br>• Yes, I received information, but in my opinion incomplete<br>• No, I have not received any information<br>• I do not remember
**(45) Was insertion of the bladder catheter painful?**
• Was not<br>• No, but it was unpleasant<br>• Yes, it hurt a bit<br>• Yes, it hurt a lot<br>• I do not remember<br>• Not applicable
**(46) Could an accompanying person be present during the caesarean section?**
• Yes, they could have been present<br>• Yes, they could, but was not present<br>• They could not, the staff did not agree, despite my request<br>• I do not know, I did not talk to staff about it<br>• Not applicable
**(47) Did you have contact with your child immediately after the cesarean section?**
• Yes, the child was put close to my face<br>• Yes, the child was on my breast<br>• No, the child was taken away immediately<br>• Not applicable
**(48) Was the child provided with kangaroo care by a loved one?**
• Yes<br>• No<br>• I do not know/I do not remember

(49) When was the first time you tried to breastfeed your baby?

- Shortly after the caesarean section in the post-operative room
- After moving to a maternity ward
- In the following days due to the mine/child's health condition
- I did not want to breastfeed my baby
- I do not remember

(50) Did you receive information on how to care for the wound after the cesarean section?

- **Yes, I have received enough information**
- **Yes, I received information, but in my opinion incomplete**
- **No, I have not received any information**
- **I do not remember**

(52) During the cesarean section did the medical staff:

- Show you respect
- Care for your privacy and intimacy
- Communicate with you in a cultural and well-mannered way
- Gave you all the needed information
- Gave you the information so that it was understandable

- Definitely yes
- Rather yes
- Neither yes nor no
- Probably not
- Definitely not

(150) Did the medical Staff in the postoperative room:

- Take care of your comfort and convenience
- Take care of pain relief
- Help in breastfeeding the child
- Help in ensuring contact with the child
- Respond to your requests and questions

- Definitely yes
- Rather yes
- Neither yes nor no
- Probably not
- Definitely not

PAGE 6 – AFTER DELIVERY

QUESTION CONTENT

(53) Was there any direct access to the bathroom in the room where you were lying after giving birth?

- Yes
- No

(54) Was your intimacy respected during medical rounds?

- Definitely yes
- Rather yes
- Probably not
- Definitely not
- It's hard to say

(106) Why do you feel that your intimacy was not respected during the medical rounds? (you can select more than one answer)

- Other women were able to observe the examination
- Other women have heard conversations with the staff
- Visitors were not asked to leave
- Too many people participated in the medical rounds
- I did not have the opportunity to prepare myself for the medical rounds
- Another reason, which?
- It's hard to say

(55) During your stay in the maternity ward, could you be with your child without restrictions?

- Yes, the baby's cot was next to my bed
- Yes, I could visit the baby all the time
- Did not

(56) Child related procedures such as test, vaccinations etc. were carried out in your presence?

- **Yes, all**
- **Yes, but only some**
- **Were not**

(107) During these tests, could you (more than one answer possible):

	• Only accompany your child<br>• Have the child in your hands<br>• Have the child breastfeed<br>• I do not know / I do not remember
**(57) Did the maternity ward staff help and teach you how to care for the child?**	
• Yes, the staff did it on their own initiative<br>• Yes, but I had to ask for it<br>• No, but I did not need help<br>• No, although I needed help	
**(58) Were the child care treatments (such as changing diapers, bathing) performed in your presence?**	
• Yes, all<br>• Yes, but only some<br>• Do not	
**(59) Did the maternity ward staff help you and teach you how to breastfeed your baby?**	
• Yes, the staff did it on their own initiative<br>• Yes, but I had to ask for it<br>• No, but I did not need help<br>• No, although I needed help	
**(64)<br>Was it possible to use the services of a lactation advisor in the ward?**	
• Yes, all the time<br>• Yes, but only at designated times<br>• Was not<br>• I do not know	
**(65) Was your child given formula (modified milk) in the hospital?**	
• Yes, for medical indications<br>• Yes, at my request<br>• Yes, without informing me<br>• Was not<br>• I do not know	
**(60) How do you assess breastfeeding support (1 is not enough - no support, 5 - very good support)**	
Scale 1-5	

(61) Were you asked for consent regarding:
• **Newborn bath**<br>• **Vaccination of a newborn baby**<br>• **Newborn examination**<br>• **Feeding a newborn baby with modified milk**<br>• **Drug administration**<br><br>• **Yes**<br>• **Was not**<br>• **I do not know**<br>• **The child did not require such a procedure**
**(62) Could a relative stay in the maternity ward?**
• Yes, all the time<br>• Yes, during visiting hours<br>• Yes, with some limitations<br>• No, it was not possible to be near relatives
**(63) Did the maternity ward staff:**
• Show you respect<br>• Care for your privacy and intimacy<br>• Communicate with you in a cultural and well-mannered way<br>• Gave you all the needed information<br>• Gave you the information so that it was understandable<br><br>• Definitely yes<br>• Rather yes<br>• Neither yes nor no<br>• Probably not<br>• Definitely not<br>•
**(152) Please, evaluate the conditions after giving birth in the following categories (1 is not satisfactory, 5 very good):**
• cleanliness<br>• Standard of equipment (eg cabinets, beds)<br>• Room standard (e.g. bathroom, size)<br>• Aesthetics of the department (eg decor, interior colors)<br>• Food quality

PAGE 7 – STAFF – INFORMATION, SUPPORT

QUESTION CONTENT	
**(69) In the hospital, did you feel sufficiently informed about:**	
● Childbirth<br>● Your health<br>● Health of the child during delivery<br>● Newborn's health<br>● How to feed a child<br>● Procedures performed to the child<br>● Who from the staff looks after you<br><br>● Definitely yes<br>● Rather yes<br>● Neither yes nor no<br>● Probably not<br>● Definitely not	
**(70) Do you feel that any of your rights were violated during your stay in the hospital?**	
● Yes<br>● No<br>● I do not know	
	(108) Did you write a complaint regarding this case (eg to the hospital management, a medical or midwifery organisation, Patient Rights Representative)?
	● Yes<br>● No
**(71) Have you experienced any of the following situations in the hospital (please mark all the situations that happened to you)?**	
**● I had to ask repeatedly to change the bedding**<br>**● Despite calling, I had to wait long for the arrival of someone from the staff**<br>**● I did not have anyone to leave my child with while using the shower**<br>**● I have not received the necessary support in feeding the child**<br>**● I have not received the necessary support in dealing with breastfeeding, breast wounds**<br>**● I have not received the necessary support in dealing with a lowered mood**<br>**● An accompanying person was not allowed to be with me during the entire delivery**<br>**● I felt uncomfortable about people visiting other women**<br>**● The toilet / bathroom was dirty**<br>**● I did not have access to the fridge, kettle**<br>**● A payment for delivery with an accompanying person was required**	

● **None of the above situations took place**
**(72) Have you ever had a staff member in the hospital (please indicate all the situations that you remember):**
● Forced fees<br>● Blackmailed you with your or your child's health<br>● ridiculed<br>● threatened<br>● prodded<br>● slapped<br>● Flexed your legs during pushing<br>● Tied your legs to the delivery bed<br>● Another unpleasant situation, which one?<br>● I have not experienced any of the above situations

PAGE 8 – STAFF – INTIMACY, RESPECT, COMMUNICATION

QUESTION CONTENT

(73) Do you feel that any activity in the hospital has been done:

- **Not gently enough**
- **Without your permission**
- **Without care for your privacy or intimacy**

- **Yes**
- **Did not**
- **It's hard to say**

(155) Which of these activities was performed, according to you, not gently enough?

- Internal / gynecological examination
- Insertion of a vein canula
- Enema
- Episiotomy
- Crotch stitching
- Removal of stitches after crotch sewing
- Insertion of a bladder catheter
- Insertion of a tablet / suppository
- Latching the child to the breast
- Other, what?

(156) Why did you feel that the staff did not take care of your privacy or intimacy?

- Leaving open, unclosed doors
- Too many staff members during conversations and surveys
- Too many students during tests and delivery
- Conversations, tests in the presence of other patients in the room
- Conversations, tests in the presence of visitors
- Another reason, which?

(74) Please remember how the staff spoke to you. Do you feel that during your stay in the hospital someone from the staff:

- Has used incomprehensible language
- Has raised their voice / shouted at you
- Has insulted you
- Has said inappropriate comments about your person, your situation
- Has not introduced herself/himself
- Has not answered your questions
- Has addressed you in the third person, eg let her undress
- Has addressed you in a confidential manner
- Has exalted herself/himself, has treated you from above
    - Yes
    - No

	(114) Please describe the situation when the staff used incomprehensible language.
	(115) Please describe the situation when staff raised their voices / shouted at you.
	(116) Please describe the situation when the staff insulted you. With what words / wordings the staff has offended you? Please, write out all that you remember.
	(117) Please describe the situation when the staff said inappropriate comments about your person, your situation.
	(118) Please describe the situation when the staff mocked you. What words / wordings of staff have offended you? Please, write out all that you remember.
	(120) Please describe the situation when the staff did not answer your questions.
	(147) Please describe the situation when the staff addressed you in the third person. What words / wordings of staff have offended you? Please, write out all that you remember.

	(148) Please describe the situation when the staff addressed you in a confidential manner
	(121) Please describe the situation when the staff was exalted, treated you from above

(157) During your stay in the hospital, did the hospital staff introduce themselves?

- Yes, always
- Yes, often
- Yes, but only sometimes
- Yes, but rarely
- They did not introduce themselves at all

(75) When remembering your stay in the hospital, do you feel that for whatever reason you were treated worse than other patients?

- Yes
- No

(76) Do you think that you were treated worse because of:

- Your age
- Your state of health
- Your financial situation
- Your nationality
- Your religion
- Your body weight
- Your previous preparation for delivery (eg homebirth)
- Directing requests to staff
- The way of conceiving a child (in vitro)
- Other

- Yes
- No

(124) What is your religion?

	• Catholic<br>• Orthodox<br>• Protestant<br>• Islam<br>• Judaism<br>• Other<br>• I would prefer not to answer
	(125) What is your nationality?
	(159) For what other reason did you feel that you were treated worse?

PAGE 9 - PAYMENTS

QUESTION CONTENT

(77) During the stay in the hospital was there a situation when you were forced to do something by the staff?

- **Yes**
- **Was not**
- **I do not remember**
- **It's hard to say**

(126) What did you feel forced into while in hospital?

- Vaginal delivery
- breast-feeding
- Feeding the child with formula milk
- Vaccination of a newborn baby
- Newborn's baths
- Paying a fee
- To something else, what?
- It's hard to say

(132) Did the behavior of the staff or the way care was organized the hospital made you feel:

- **Ashamed**
- **Ignored**
- **Ridiculed**
- **Humiliated**
- **I did not feel any of the above emotions**

(79) Have any fees been associated with the stay in the hospital?

- Yes
- No

(127) If there were any fees associated with childbirth - what did you paid for. Please tick all the appropriate answers.

	• Childbirth with an accompanying person<br>• A separate room for delivery<br>• Presence of the chosen midwife at delivery<br>• Presence of the selected doctor at delivery<br>• Single room after delivery<br>• Epidural anesthesia<br>• Childbirth in water<br>• Donation, "brick"<br>• Other fees
	(138) Please enter the amount you paid for the birth with an accompanying person:
	(139) Please enter the amount you paid for the delivery in a single room:
	(140) Please enter the amount you paid for the presence of the chosen midwife at birth:
	(141) Please enter the amount you paid for the presence of your chosen doctor at birth:
	(142) Please enter the amount you paid for the single-person room after delivery:
	(143) Please enter the amount you paid for an epidural:
	(144) Please enter the amount you paid for childbirth in water:

	(145) Please enter the donation amount you paid to the hospital:
	(146) What other fees did you have to pay in the hospital due to childbirth?

(80) A good birth is in your opinion (please enter the first three associations):

(81) Please select the expression that is closest to your childbirth experience:

- Childbirth is an ecstatic experience, a source of great pleasure and a sense of power
- Childbirth is a positive experience
- Delivery, like delivery, you just have to go through it
- Childbirth is a negative experience
- Childbirth is a traumatic experience, the most horrible thing I've ever experienced

(67) If you were to give birth again, would you choose this hospital again?

- Definitely yes
- Rather yes
- Neither yes nor no
- Probably not
- Definitely not

(68) How would you rate the care you received in the hospital on a scale of 1-5 (1 is not enough, 5 very good)

(88) Would you like to recommend to other women the midwife from the hospital where you gave birth? Please enter the name of the recommended midwife.

(154) Would you like to recommend to other women a doctor from the hospital where you gave birth? Please enter the name of the recommended doctor.

**(153) Additional comment:**

PAGE 10 – DEMOGRAPHY

QUESTION CONTENT
**(83) Size of the place of residence**
• City above 500 000 inhabitants<br>• City above 100 000 – 500 000 inhabitants<br>• City 50 000 – 100 000 inhabitants<br>• City up to 50 000 inhabitants<br>• Village
**(84) Your year of birth:**
**(85) Education:**
• Primary / lower secondary<br>• Basic professional<br>• High School<br>• Post-Secondary / Vocational<br>• Higher (bachelor, master, engineer)
**(86) How do you assess your financial situation?**
• Very good<br>• Good<br>• Average<br>• Bad<br>• Very bad<br>• I would prefer not to answer
**(136) Do you suffer or have suffered from any of the following diseases?**
• Hepatitis B<br>• HIV / AIDS<br>• Psoriasis<br>• Cancer<br>• Obesity<br>• Mental disorder<br>• I am disabled with mobility<br>• I did not get sick and I do not have any of the above illnesses

- I would prefer not to answer
